# Effect of Augmented Reality (AR) Models on Patient Education in Preventing Non-steroidal Anti-inflammatory Drug (NSAID)-Induced Acute Kidney Injury

**DOI:** 10.7759/cureus.69265

**Published:** 2024-09-12

**Authors:** Nirmay Sonar, Shohan Pervaze, Gurvinder Kaur, Zaynah Sadiq, Sathiyendram Poobalasingham, Bernie Sergent

**Affiliations:** 1 Internal Medicine, Norton Community Hospital, Norton, USA; 2 Medicine, Mahatma Gandhi Mission Institute of Health Sciences, Navi Mumbai, IND; 3 Nephrology, Bristol Regional Medical Center, Bristol, USA

**Keywords:** acute kidney injury, augmented reality, general nephrology, information quality, nephrology, nsaid induced nephritis, patient education

## Abstract

Acute kidney injury (AKI) is a prevalent condition, particularly affecting critically ill patients. Nonsteroidal anti-inflammatory drugs (NSAIDs) are a significant contributor to this condition. Traditional educational methods often fail to effectively convey the risks associated with NSAID use. This study investigated the application of augmented reality (AR) models to enhance patient education in NSAID-induced AKI within an outpatient clinic. The study, conducted over four months with 67 participants, employed a questionnaire-based approach to assess knowledge levels before and after exposure to AR models. The results demonstrated a substantial increase in patient knowledge, and a majority of the participants expressed a willingness to modify their behavior to safeguard their kidneys. The findings suggest that AR holds enormous potential in improving patient comprehension and promoting positive health behaviors. However, limitations such as variations in patient education levels and technology proficiency were acknowledged. Further research is necessary to explore the long-term impact of AR-based education in broader clinical settings.

## Introduction

Acute kidney injury (AKI) is a common and serious condition, with a global prevalence ranging from 1% to 25%, depending on the setting and patient population [1]. Critically ill patients are particularly vulnerable, with prevalence rates exceeding 20% [2]. Nonsteroidal anti-inflammatory drugs (NSAIDs) are a widely used class of medications known to contribute to AKI, with studies showing an odds ratio of 1.73 for NSAID-induced AKI [2,3]. NSAIDs causing kidney injury are multimodal, decreasing the afferent arteriole perfusion, causing direct tubular injury, and impairing the ability for fluid excretion [4]. Data on outpatient NSAID-induced AKI is variable, with some estimates for the prevalence ranging from 0.5-2% [5].

Patient education on the risks associated with NSAID use is crucial, but traditional methods such as pamphlets often fall short of achieving the desired level of understanding. Education stands as the cornerstone of effective patient care. The principles of 4E’s framework guided the research, focusing on educating patients, enhancing understanding through augmented reality (AR) models, evaluating knowledge through questionnaires, and measuring the effect on patient behavior.

This study investigates the potential of AR models as an educational tool in an outpatient clinic setting. A recent study in 2023 explored the use of AR models in intracranial aneurysms and found that patients were better able to understand their condition [6]. AR models have a tremendous capacity for closing gaps in health literacy [7]. By harnessing the power of visual learning, which has proven to enhance retention and comprehension, the study posits that AR can substantially enhance patient knowledge and ultimately lead to improved healthcare outcomes.

## Materials and methods

The study was conducted over a four-month period in the outpatient clinic of Norton Community Hospital, an internal medicine residency clinic in Norton, VA. The principles of 4Es (educate, enhance, evaluate, and effect) were the framework of this study. The objectives of this study were to evaluate the effectiveness of augmented reality models in enhancing patient knowledge about NSAID-induced AKI, to compare the pre- and post-intervention knowledge scores among patients exposed to the AR model (intervention being exposure to the AR models), and to assess the impact of AR-based education on patient behavior regarding NSAID use.

The study included adults aged 18-60 years. Exclusion criteria were dialysis-dependent kidney disease or end-stage renal disease (ESRD), unwillingness to participate, legal blindness, and hearing impairment. Initially, 100 participants were targeted, and a total of 67 completed the study. The sample size was based on a balance between statistical power and feasibility within a four-month period in a community hospital setting. The sequential, time-bound sampling approach was employed to represent the clinic’s patient population, thereby ensuring the generalizability of the findings to comparable outpatient settings.

A questionnaire-based format was used for data collection. This questionnaire was developed clinically with the input of physicians with experience in nephrology and patient education. We specifically developed this questionnaire for this project to address specific knowledge gaps, ensuring a clear and succinct understanding of questions and options. Participants completed a pre-intervention questionnaire assessing their baseline knowledge of NSAIDs and kidney function. Following exposure to the AR model (which is the intervention), a post-intervention questionnaire measured changes in knowledge and behavior. The maximum possible score on the questionnaire was nine. Table 1 illustrates the questions used for this purpose with pre- and post-intervention questionnaire sections.

**Table 1:** The questionnaire was used to assess pre- and post-intervention and calculate knowledge scores Intervention refers to the exposure to the augmented reality (AR) model. The questionnaire is a multiple-choice type; common brand names are used in question 1 for patient understanding. Every correct answer rewards one point on the knowledge score. Pre-intervention questionnaire: correct answer selections: Question 1: a, b, and c; Question 2: a, b, and c; Question 3: a, b, and c; Question 4 does not contribute to the score, it is only for patient education. Post-intervention questionnaire: correct answer selections: Question 1: a, b, and c; Question 2: a, b, and c; Question 3: a, b, and c; Question 4 does not contribute to the score. Question 5 is a behavioral change question that assesses the response to intervention and future change. Question 6 is for feedback regarding the model. NSAIDs: non-steroidal anti-inflammatory drugs; mg: milligrams

Pre-intervention questionnaire	Options (mark all that apply)
1. Select the NSAIDs you know	a. Acetaminophen (Tylenol)
	b. Ibuprofen (Motrin)
	c. Naproxen (Aleve)
	d. None of the above
2. What is the role of kidneys in our body?	a. Filtration of blood
	b. Excretion of toxins
	c. Detoxification of wastes
	d. None of the above
	e. Not sure
3. How Do NSAIDs affect your kidney?	a. Decrease blood flow to the kidney
	b. Cause damage to kidney structure
	c. Affect urine flow
	d. None of the above
	e. Not sure
4. Safe dose of Acetaminophen/Tylenol in a day?	a. 3250 mg
	b. 6000 mg
	c. 1250 mg
	d. Not sure
Post-intervention questionnaire	Options (Mark all that apply)
1. Select the NSAIDs you know	a. Acetaminophen (Tylenol)
	b. Ibuprofen (Motrin)
	c. Naproxen (Aleve)
	d. None of the above
2. What is the role of kidneys in our body?	a. Filtration of blood
	b. Excretion of toxins
	c. Detoxification of wastes
	d. None of the above
	e. Not sure
3. How do NSAIDs affect your kidney?	a. Decrease blood flow to the kidney
	b. Cause damage to kidney structure
	c. Affect urine flow
	d. None of the above
	e. Not sure
4. Safe dose of Acetaminophen/Tylenol in a day?	a. 3250 mg
	b. 6000 mg
	c. 1250 mg
	d. Not sure
5. Since learning about NSAID-induced acute kidney injury through the augmented reality model, have you made any changes to your medication usage or habits to better protect your kidney health?	a. Yes, I have reduced my NSAID usage or switched to alternative pain relief methods.
	b. No, I have not made any changes to my medication usage or habits.
6. Rate the model from 1-5 in terms of how it has helped you in terms of education	1 - Not helpful
	2 - Somewhat helpful
	3 - Not sure
	4 - Helpful
	5 - Very helpful

Participants were shown a 3D AR model that illustrated the role of kidneys and how NSAIDs can induce AKI. The model was viewed on widely available devices such as smartphones and tablets. This is also the intervention of the study. Interactive Model 1 is a three-dimensional rendering of the augmented reality model used in the study.

**Video 1:** AR kidney model used in this study AR: augmented reality

Providers were instructed to educate patients in lay language, the basic understanding of kidney structure and function, with animation depicting the flow of blood, the processes with a simple cycle animation, and the outward excretion of urine. The provider then educates on the mechanisms of NSAID-induced kidney injury in simple terms such as limitation to blood flow, damage to kidney tissue, and decreasing urine output. Video 1 demonstrates a simulation of the animation used in the model, which was shown to the patients.

**Video 1:** Animation demo using the augmented reality (AR) model

Pre- and post-intervention scores were compared to assess the effectiveness of the AR model. The mean pre-intervention score was 3.52±1.29, and the mean post-intervention score was 6.52±1.74. Statistical analyses included the calculation of relative and absolute changes in knowledge scores, Cohen’s D, T score, and p-value.

## Results

The study demonstrated a significant improvement in patient knowledge following the AR intervention. The mean pre-intervention score was 3.52±1.29, while the mean post-intervention score was 6.52±1.74. The lowest pre-intervention score was one, and the highest was nine. The relative change in knowledge score was 1.07, and the absolute change was 3.0±1.41. 

**Figure 1:**
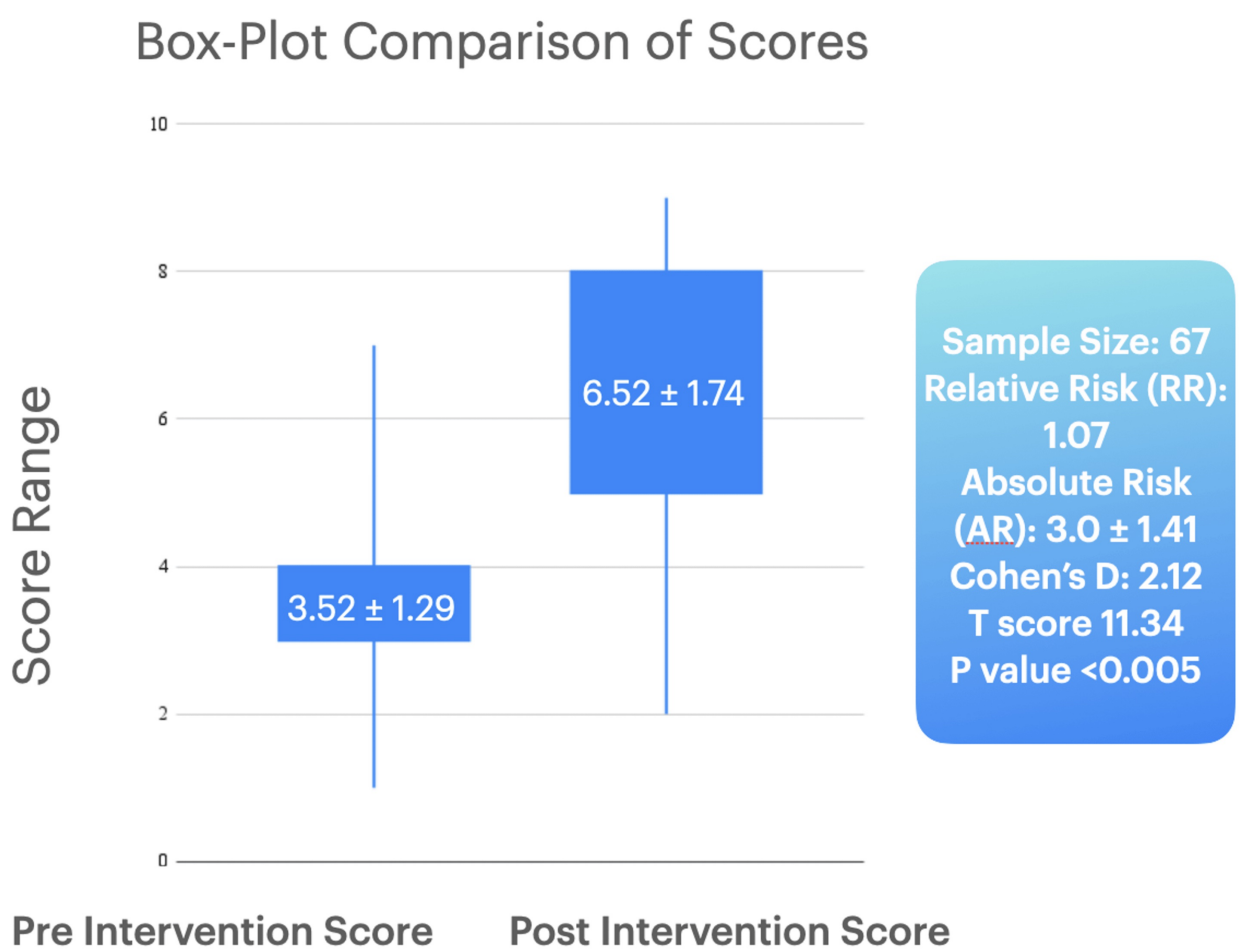
Box plot comparison of pre- and post-intervention knowledge scores with additional statistic measures in the adjacent box Score refers to pre- and post-intervention knowledge scores. As mentioned in the materials and methods sections, this refers to responses to the questionnaire before the intervention, and then post-intervention. The intervention refers to exposure and learning through the augmented reality model.

A Cohen’s D of 2.12, which expresses a large effect size, indicating that the intervention had a substantial effect on the knowledge scores, as well as a T score of 11.34 with a p-value of <0.005, which indicated a statistically significant difference. Figure 1 shows a box plot comparison of the score comparison in respect to the sample size used.

**Figure 2:**
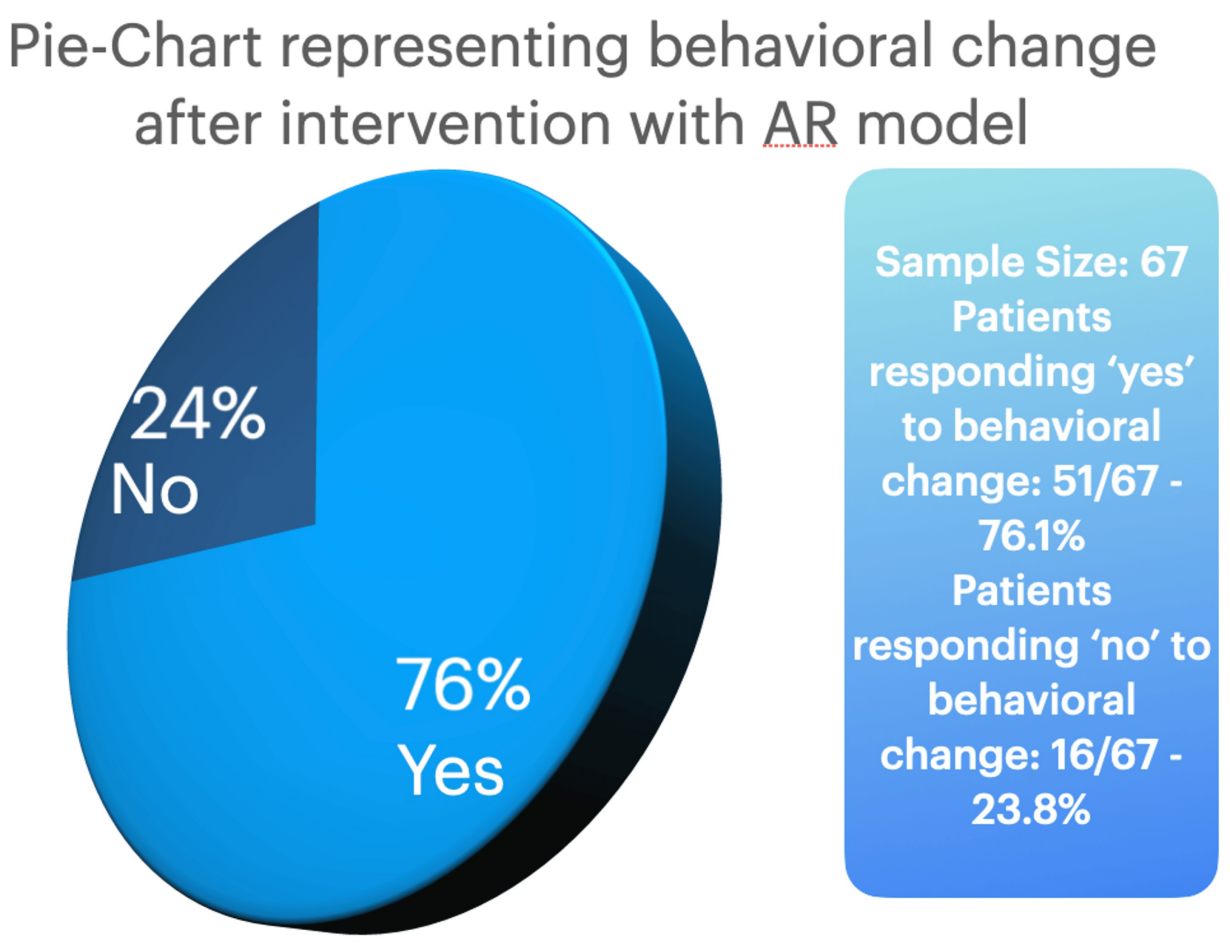
Pie chart representing responses to the behavioral change question AR: augmented reality

Additionally, a total of 51 out of the 67 participants responded that they would be willing to make changes to protect their kidneys from renal injuries caused by NSAIDs, which is an estimated 76.1% of the sample size, while 16 out of 67 responded they would not. Additional behavioral analysis, including what limits patients in making the change, and techniques such as motivational interviewing were used; however, recording of those responses was performed as a part of the medical outpatient visits and was not a part of the results. Figure 2 shows a pie chart representing the prevalence of behavioral change after the AR model intervention.

As part of the study’s evaluation, an assessment was conducted to evaluate the patient’s perception of the model’s educational value. This assessment was included as question six on the post-intervention questionnaire. The majority of the responses indicated a rating of four out of five, which corresponds to the term “helpful.” Table 2 represents a tabular representation of the data in the study.

**Table 2:** Measured outcomes of the study in tabular form Intervention refers to exposure to the AR model in the study. Pre-intervention knowledge score is calculated before the exposure to the model using the pre-intervention questionnaire. Post-intervention knowledge score is calculated after the exposure to the model using the post-intervention questionnaire. AR: augmented reality

Measure	Result
Sample size	67
Mean pre-intervention knowledge score	3.52±1.29
Mean post-intervention knowledge score	6.52±1.74
Relative risk (RR) or relative change in knowledge score	1.07
Absolute risk (AR) or absolute change in knowledge score	3.0±1.41
Cohen’s D	2.12
T score	11.34
Patients responding yes to behavioral change question	51 out of 67
Patients responding no to behavioral change	16 out of 67

## Discussion

The primary directive of this study was to evaluate the effectiveness of AR models in enhancing patient education in NSAID-induced AKI. The results strongly suggest that AR is a valuable tool in improving patient understanding and promoting positive health behaviors. Most providers in this study, while conducting the intervention, reported that the longer patients spent interacting with the model, the more frequently they would take the initiative to engage with it, the more favorable the results they observed, and the deeper their understanding of the subject. The results correlate with an improvement in patient knowledge and an improvement in patient behaviors, which reinforces that education stands as the cornerstone of patient care. The AR model facilitated a deeper comprehension of complex medical concepts, contributing to higher retention and application in real-life scenarios [6,7]. In consultation with nephrology experts, this project has the potential to yield significant real-world implications. For instance, it could serve as an educational tool during screening and health camps, enabling patients to comprehend the intentions of healthcare providers [8,9]. Another application where the education and AR models system would prove beneficial to patients is in the context of dialysis and end-stage renal disease. Patients often harbor reservations and apprehensions about dialysis, and patient education through visual learning can facilitate improved decision-making and a comprehensive approach to care.

However, several limitations were identified. The subjective nature of the scales employed to assess knowledge and the variability in patient education levels and technology proficiency were potential sources of bias. Patient education levels vary, and responses based on patient stratification could be explored in future studies. The questionnaire utilized, while clinically developed, lacks standardization. It was collaboratively created with nephrologists to evaluate patient understanding within a clinical setting. Furthermore, while technological advancements are innovative, they may not be as reliable as conventional methods in all circumstances. For instance, in situations where technology is not readily available or experiencing technical interruptions, conventional methods may be more suitable [7,10]. In 2019, a study employed a five-point Likert scale to compare conventional 3D printed models with augmented reality models. The study revealed that patients exhibited a greater understanding of the subject matter when exposed to physical models [11].

The current literature on AR models in renal injury is limited. A recent scoping review conducted in 2020 identified applications where AR models were found to be a robust teaching tool for providers. The study also highlighted the potential of AR models for patient education [7]. Another review underscores the need for additional high-quality study designs to fully comprehend the value of AR in patient education [12].

## Conclusions

This study highlights the potential of augmented reality as a potent tool in patient education, particularly in the context of NSAID-induced acute kidney injury. By addressing the limitations and integrating AR into clinical workflows, healthcare providers can enhance patient engagement and improve health outcomes. Future studies should investigate the long-term impact of AR-based education and its applicability in broader clinical settings.
